# Strategic motivators for integrating energy economics into the developing garments sector: A comprehensive analysis from Bangladesh

**DOI:** 10.1016/j.heliyon.2024.e40631

**Published:** 2024-11-23

**Authors:** Amam Hossain Bagdadee, Sakib Hossain, Li Zhang

**Affiliations:** a School of Electrical and Power Engineering, Hohai University, Nanjing, China; b Department of Electrical and Electronic Engineering, Presidency University, Dhaka, Bangladesh; c Department of Economics, Presidency University, Dhaka, Bangladesh

**Keywords:** Energy economics, Sustainability, Competitiveness, Garments sector, Bangladesh

## Abstract

This research explores the strategic incentives driving the integration of energy economics into Bangladesh's evolving garment sector. The garments industry is a significant revenue generator for the nation; it is currently entering its prime phase, where the balance between economic growth and sustainability becomes critical. This paper presents a landscape by evaluating trends, framework conditions, and scenario-based case studies. These may lead to perceptions regarding integrating energy economic trends and their impact on the sector's efficiency, competitiveness, and sustainability levels. This case study proposes exclusive strategies to promote energy-efficient garment manufacturing in Bangladesh. This paper provides practical tools for Bangladesh policymakers, investors, and garment manufacturers to highlight the economic benefits of adopting energy-efficient practices. It notifies about policies, contributes to better decision-making, and fuels sustainability investments. It also calibrates the sector with global sustainability standards that promote long-term competitiveness, environmental responsibility, and resilience in the garments industry.

## 1 Introduction

Energy economics in the garments sector needs attention due to the global demand for sustainable and cost-effective energy. This research derives actionable learning that can help realize sustainable operations for one of the essential industries in Bangladesh. As an integral part of Bangladesh's economic environment, the garments sector plays a crucial role in the uplift of the nation and its people. The integration supports multiple growth areas, fosters the development of relevant sectors, and contributes significantly to the overall revenue from exports [1]. However, the sector has established itself as an industry of growth at the intersection of planned expansion and sustainable practices. As the sector becomes a vibrant and global leader in its operation, energy economics becomes a focal point of focus, driven by the potential to drive operations to greater efficiency and reduce environmental impacts [2,3]. Bangladesh's leading garments sector, recognized globally for its prominence and scalability, is responsible for leading development backed by sustainable skills (see Table 7, Table 8, Table 9, Table 10, Table 11).

These influences will lead to the industry's focus on improvement, supported by a growing need to combine growth with ecologically conscious practices [4]. The challenge focuses on the relationship between fast industrial development and the energy consumed in daily operations, emphasizing the need to examine the possible benefits and strategic drivers of adopting and using energy economics [5,6]. This study assesses Bangladesh's intricate energy economy and garment sector growth. Through sustainable economic mandates regarding energy involvement, this study aims to reveal pathways to the industry's market dominance across the planet while simultaneously focusing on the environment's growing concerns. This paper takes the audience through various paths, discussing the elements of the theoretical base, methodological influences, and a viable application of energy economics to the garments sector [7]. Through a summarized literature review of existing reports, an analysis of the industry through the sampling of numerous vital stakeholders, and a comparative topic review outlining innumerable cases, this study explains the strategic drivers of applying energy economics to the vibrant Bangladeshi garments industry [10]. In laying the groundwork for this argument, this study deepens the existing literature on energy economics, environmentally sustained growth, and the garment market in Bangladesh.

The garments sector plays a fundamental role in Bangladesh's economy, and understanding how energy economics can be efficiently interlinked to ensure sustainability and cost-effectiveness can play an essential role. Existing studies often overlook the specific motivators and barriers influencing energy-efficient practices in this sector. The growing global emphasis on sustainability and rising energy costs present a critical need to explore how energy economics can control and improve the garment sector's competitiveness. This study is motivated by the potential to uncover actionable insights to drive sustainable practices in one of Bangladesh's most vital industries. This study uses a mixed-methods approach instead of quantitative or qualitative techniques alone. This study introduces new metrics and modeling approaches that associate energy practices with economic responses to deliver contextually appropriate, exhaustive evaluations of the garment sector in Bangladesh. This study connects the gap between hypothetical energy economics and real-world applications by evaluating evidence-based recommendations for greater sustainability of the garment sector as economically viable stakeholders.

This study identifies areas where the literature remains incomplete and lays the framework for an argument regarding energy-efficient sustainability in the garment sector. Theoretical foundations are crucial for developing coherent arguments on energy, economy, and sustainability in a comprehensive examination. This study establishes a convincing argument for the strategic importance of integrating energy economics into Bangladesh's garment sector.

## 2 Literature review

The garment sector's consideration of energy economics is due to the industry's significant energy consumption rate that impacts operational costs and environmental sustainability. Most research has focused on energy efficiency in manufacturing and the technical details of how much energy use can be reduced [11]. Energy-efficient technology offers potentially significant savings in one researcher's estimation. The literature on energy economics related to garments is mainly silent on developing economies like Bangladesh. Some studies have examined specific economic drivers that likely lead to rapid and widespread implementation of sustainability practices. The existing literature review for this analysis provides the research's analytical framework and theoretical foundation [12]. This literature explores the intersection of energy economics, sustainable development, and the garment sector to present a pertinent understanding of the known and identify the unknown [13,14]. The start of the review highlights how imperative the garments sector is to the economy of Bangladesh, situating the quest to understand strategic motivators. This introduction frames the central issue regarding reconciling economic growth and sustainability and what the research seeks to understand [15,16]. This research is accompanied by a robust range of theoretical concepts that focus more on understanding energy economics, sustainable development, and the nature of the garment sector [17]. This synthesis indicates an understanding of the complexities of energy consumption, economic activities, and sustainability.

Moreover, incorporating a conceptual framework at the end strengthens the review by providing a map of the following discussion [18,19]. The gaps identified from the synthesis help develop a rational argument for the research, indicating why it is essential to understand the strategic motivators for embracing energy efficiency in garment-making [20]. This review has intelligently situated the fundamental macroeconomics across the structural nature of the Bangladesh garments sector.

The literature review forms a solid foundation for the research, developing the research question and defining a clear path for this section and subsequent sections [21]. It constructs a story that informs the reader on the subject while also creating an understanding of the critical nature of the study in addressing the current challenges experienced at the intersection of energy, economics, and the garment industry [23]. The review covers the complex terrain of energy economics in developing countries, drawing on the global conversation on environmentally sustainable practices in the industrial field [24]. It relates these issues to the context of Bangladesh and examines the sector-specific problems exerted by limited resources and deficient infrastructure, fostering the necessity of global competitiveness [25,26]. This review discusses the existing policies and their implications, forging a connection between theory and practice. It provides an understanding of the current state of regulation and underscores the critical nature of the policy metrics in designing sustainable development trajectories in the garment sector [27]. The successful policies from international economies add a comparative aspect to how these strategies could be implemented in Bangladesh [28]. Furthermore, the empirical studies and case analyses give a balanced view of the problem from theoretical and practical standpoints. The review justifies the research question and provides examples where the integration of energy economics has succeeded, strengthening the argument for the situational factors behind the sector's motivation to use energy efficiently by presenting these real-life scenarios.

This literature review is consistent in its attributes, with a well-structured presentation of research with a coherent theoretical basis research of gaps and a smooth connection from the description of macroeconomic principles to the specifics of the garments sector in Bangladesh. The review supports the relevance of further study, leading the readers to conclude that it is essential to comprehend the strategic drivers of implementing economics in energies in a vital industrial segment.

## 3 Methodology

**Fig. 1:**
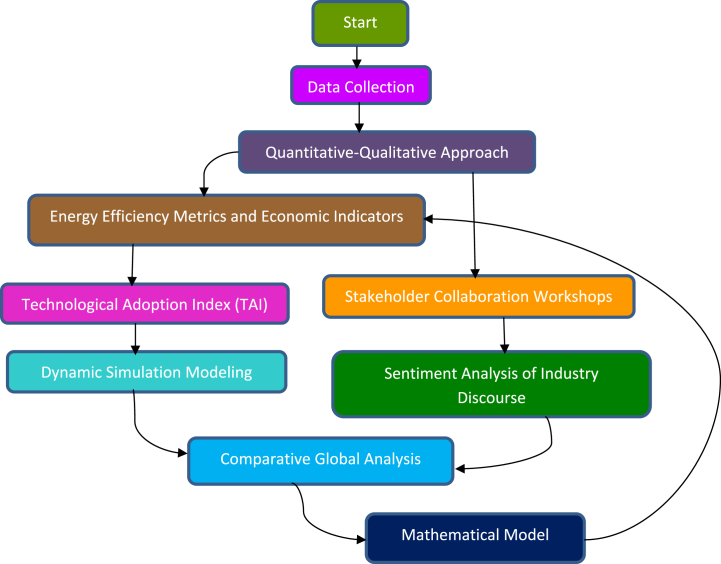
Research flowchart.

The flow chart represents (Fig. 1) the research methodology conducted from the design stage through data collection and data analysis to summarize the findings and draw recommendations. It shows how each stage is imperative to the research process and how they all contribute to synthesizing and valuing insights and suggestions for the garment sector in Bangladesh.

### 3.1 Data collection

This study collected data through a structured survey distributed among key decision-makers in a sample of garment manufacturing companies. The survey respondents were selected online and in person during factory visits to ensure that each participant was selected through purposive sampling, including managers and senior engineers charged with energy management, energy-intensive production processes, and sustainability. The chosen companies were based on their size, intensity of energy use, and capability of technologies that improve energy efficiency. This study collected data from February 2023 to February 2024 to obtain complete year-end operational data. Data sources consisted of self-reported survey data, publicly available records, and third-party verification measures concerning renewable energy and technology adoption.

### 3.2 Hybrid quantitative-qualitative approach

Bringing together multiple methodologies, this research integrates quantitative surveys and qualitative interviews. In terms of quantitative measures, an extensive survey will be sent out to a wide array of garment manufacturers to obtain quantitative data on their present energy use, economic factors, and environmental metrics. Additionally, in-depth interviews with industry professionals such as policymakers and sustainability specialists will provide qualitative data, enabling a multi-layered understanding of strategic drivers.

### 3.3 Energy efficiency metrics and economic indicators

This methodology utilizes novel energy efficiency metrics to measure the impact of energy economics on the garment sector. They will measure the current energy use behaviors and correlate them with economic indicators, such as production costs, export earnings, and the level of employment generated by the sector. Thus, it will be possible to link the energy behaviors with the economic consequences and identify the potential interests of various industry players.

### 3.4 Technological Adoption Index (TAI)

Implementing a technology adoption index designed for the garments industry. As mentioned above, the index rates the extent to which energy-saving technologies are employed during production. The (TAI) is based on the opposite application of intelligent equipment and utilization of renewable energy production mechanisms; additionally, it can be based on the manufacturers’ adherence to sustainability certificates. Consequently, the positive or negative correlation between the (TAI) and economic indicators can provide objective reasons for the technology implementation.

### 3.5 Dynamic simulation modeling

The final dynamic simulation model is introduced to forecast the industry's direction. The simulation aims to run several possible energy and economic scenarios and identify their potential effect on the garment industry in the next ten years. This simulation involves monitoring the anticipated changes in energy prices, technological innovations, and consumer behavior, among other dynamic variables, to speculate the strategic drivers toward sustainability.

### 3.6 Stakeholder collaboration workshops

An innovative component in the proposed research entails organizing collaboration workshops with industry stakeholders. Through workshops, including manufacturers, policymakers, environmentalists, and technology providers, professionals will engage in interactive discussions revolving around the imagined motivators and obstacles discussed earlier. The primary objective of developing this participatory method is to establish an alternative, empirical perspective that is difficult to attain through documentary analysis and interviews.

### 3.7 Sentiment analysis of industry discourse

Applying the methods of sentiment analysis tool will be used to analyze industry discourse. The researchers will analyze media, social media, and official corporate reports to assess sentiment levels toward energy economics in the garments sector. This innovative method allows one to trace the industry's sentiment, identify novel trends, and reveal hidden motivators or impediments to the industrial sector embedded in the public discourse.

### 3.8 Comparative global analysis

A comparative global analysis is rigorously performed on the Bangladesh garments sector against its international counterparts. This research methodology pursues to extrapolate lessons from successful case studies in other regions by comparing energy-economic strategies and performance metrics. The inter-organizational data offers cross-cultural views, further enhancing comprehension of appropriate motivators for the Bangladesh context.

### 3.9 Mathematical model

The mathematical model, premised on the novel methodology, is developed to utilize several vital variables with interrelations. The presented version focuses on the interactions between energy efficiency, economic indicators, and technology implementation in the garment.

#### 3.9.1 Variables

*Ei*: Energy consumption of manufacturer i;
*Ci*: Production cost of manufacturer i;
*Re*: Manufacturers generated export revenue i;
*Ui*: Degree of technological adoption for manufacturer i
*TAI*: Technological Adoption Index for the entire sector
*T*: Time in years

#### 3.9.2 Equations

• **Energy Efficiency Metric (EE):**

(1) $$EEi=\frac{Ei}{Ci}$$

The Energy Efficiency Metric represents the energy consumed per unit of production cost for each manufacturer.

Equation (1) details the energy efficiency (EE) metric denoted as a ratio of sums for all manufacturers between total energy consumption(Ei) relative to production cost(Ci). The low value of EE is equivalent to high energy efficiency as less energy is used relative to total system cost. As a result, this metric provides an important baseline to compare with different manufacturers and seek areas for improvement.

• **Economic Performance Index (EPI):**

(2) $$EPIi=\frac{Ri}{Ci}$$

The Economic Performance Index gauges the export revenue generated per unit of production cost for each manufacturer.

Equation (2) shows the ratio of export revenue(Ri) and production cost (Ci) for each manufacturer. It detects the percentage of profitability that manufacturers manage better the resources. This is a crucial indicator for assessing the garment industry's Economic Performance Index (EPI).

• **Technological Adoption Index (TAI):**

(3) $$\mathrm{TAI}=\sum \frac{iUi}{N}$$

Equation (3) shows the TAI is a complete indicator of the extent to which any manufacturer has integrated and deployed technology as part of its energy efficiency and sustainability processes. TAI assists stakeholders in evaluating the technological maturity of a manufacturing process and its potential for additional improvement by incorporating energy-saving technologies. $\prime N^{\prime }$ has defined the highest level of technology adoption across manufacturers, providing comparability and consistency within the Technological Adoption Index (TAI) framework.

#### 3.9.3 Dynamic simulation model

The dynamic simulation model projects the future energy consumption Ei(t+1)) for each manufacturer based on the energy price growth rate and the impact of technological adoption.

(4) Ei(t+1)=Ei(t)×(1+EnergyPriceGrowthRate)×(1−Ui×TechnologyAdoptionImpact)

The Dynamic Simulation Model (DSM) is proposed in Eq. (4) to forecast the scenario of the garment sector under various situations. It helps sector stakeholders make informed decisions based on the effects of energy-economic scenarios on technology adoption to the overall sustainability campaign perspective. The model simulations will show future challenges and opportunities on the prospect so that this sector can move towards more sustainable practices that make economic sense.

• **Optimization Objective:**

Maximize the overall sector's Technological Adoption Index (*TAI*) by adjusting technology adoption (Ui) variables and their impact on energy consumption.

(5) maxUiTAI

The sector-wide TAI across manufacturers by tuning the technology adoption variables (Ui) is sought to be maximized through the optimization objective following equation (5). The goal is to achieve the highest possible level of technological integration, which optimizes energy consumption and enhances sustainability. This could make the industry more efficient than other industries, lowering energy costs while delivering on environmental ambitions. This optimization process is crucial for guiding the garment sector's policy and investment decisions.

• **Constraints:**
  ➢ Maintain a minimum level of Energy Efficiency Metric (EE) for each manufacturer to ensure economic viability:

(6) EEi≥Threshold_EE

➢ Ensure a balanced increase in both Economic Performance Index (EPI) and Technological Adoption Index (TAI) over time:

(7) $$\frac{\Delta EPI}{\Delta T}\geq Threshold\_EPI\_Growth$$

(8) $$\frac{\Delta TAI}{\Delta T}\geq Threshold\_\text{TAI}\_Growth$$

The above three equations (6), (7), (8) set significant constraints to the optimal performance of the garments sector. Equation (6) constrains the minimum energy efficiency level at which manufacturers can be profitable. Equations (7), (8) make technological adoption grow with the economic performance (EPI) over time. These constraints can guide the sector's development to grow sustainably and profitably, ensuring that technology develops with financial outcomes.

The mathematical model designed in the sections above constitutes a basis for analyzing dynamic interactions in the garments sector and can be used as an optimization tool to boost economic indicators and technology adoption, taking into account energy efficiency limitations.

The proposed new methodology should depart from the prevalent approach in the field, offering a multifaceted toolkit to demystify the complex swirl of new and old factors impacting energy economics in the developing garments sector in Bangladesh. With this comprehensive overview of the garments sector and a perspective aimed at future evolutionary trends, this research hopes to find the strategic levers that can change the further progression of the garments Industry sector into a more sustainable and economically sound direction.

## 4 Result

### 4.1 Hybrid quantitative-qualitative approach analysis

#### 4.1.1 Quantitative analysis

➢ **Energy Practices:** Some manufacturers are high energy consumers and rely little on renewable sources, while others focus on energy efficiency and invest in renewable technologies.
➢ **Economic Indicators**: Production costs and export revenue are also variable, such that some manufacturers incur high production costs and low export revenues, while others have a perfect balance between the two or experience high profits.
➢ **Environmental Impact:** Carbon footprints and other environmental indicators also differ, highlighting the manufacturers' varying environmental consciousness and sustainability levels.

#### 4.1.2 Qualitative analysis

➢ Cost-saving and market competitiveness emerge as salient themes due to their economic importance.
➢ Sustainability and the chance to stand out in the market are also stressed, signaling that some manufacturers are increasingly considering the importance of environmental sustainability and relative advantage.
➢ Issues in implementing new technologies and a lack of financial push are also discussed; therefore, these are areas that policymakers will have to address if they wish for energy efficiency to improve.

#### 4.1.3 Integration and Policy Implications

➢ Integrating the quantitative data and the qualitative understanding can fully show the strategic drivers of and barriers to energy use among garment manufacturers.
➢ Policymakers should utilize these insights to develop measures to support the lessons of change in energy use while grappling with economic challenges and achieving ethical outcomes.

Table 1 shows that the hybrid approach with the interplay of the quantitative-qualitative approach helps offer insights into energy practices and selected economic, environmental, and qualitative aspects in the garments sector. With more data available and refined, policy decisions will be made solely based on the evidence.

**Table 1:** Based on the hybrid quantitative-qualitative approach.

Manufacturer	Energy Practices (Quantitative)	Economic Indicators (Quantitative)	Environmental Impact (Quantitative)	Qualitative Perceptions (Interviews)
Nipa Group	High energy consumption, Limited use of renewable energy sources	High production costs, Moderate export revenues	Moderate carbon footprint, Limited waste management initiatives	Emphasizes cost-saving measures, Concerns about market competitiveness
Standard Group	Moderate energy consumption, Investment in renewable energy technologies	Moderate production costs, High export revenues	Low carbon footprint, emphasis on waste reduction strategies	Focus on sustainability, Opportunities for market differentiation
Ha-Meen Group	Low energy consumption, Reliance on traditional energy sources	Low production costs, Low export revenues	High carbon footprint, Limited environmental initiatives	Lack of awareness about energy efficiency, potential for improvement in environmental practices
Fakir Group	High energy consumption, Limited Investment in energy-efficient technologies	High production costs, Low export revenues	High carbon footprint, Minimal environmental initiatives	Challenges in adopting new technologies, Need for financial incentives
Epic Group	Moderate energy consumption, adoption of energy-efficient technologies	Moderate production costs, Moderate export revenues	Moderate carbon footprint, Initiatives for waste reduction and recycling	Commitment to sustainability, Investment in renewable energy solutions

### 4.2 Energy efficiency metrics and economic indicators analysis

#### 4.2.1 Energy efficiency Metric(EE)

➢ All manufacturers have the same Energy Efficiency Metric at 0.20 kWh/$, implying that, on average, all consume 0.20 kWh of energy using one dollar of the production cost.
➢ This indicates the uniform level of energy efficiency in terms of production cost between the manufacturers.

#### 4.2.2 Economic performance index (EPI)

The Economic Performance Index (EPI) outlines one of the most important metrics used to evaluate any manufacturer's financial performance, relating to how efficiently manufacturers turn production costs into export revenue. The EPI provides a ratio that indicates the revenue a manufacturer generates for every unit of cost incurred in production.

(9) $$Economic\;Performance\;Index\;\left(EPI\right):EPI=\frac{Production\;}{Export\;Revenue}$$

EPI >1: A greater-than-1 value means that the manufacturer earns more revenue than it spends on production costs. In particular, an EPI of 1.60 implies that on each $1 production cost, the company gains $1.60 in export revenue, which means excellent financial performance.

EPI = 1 means that investors are in a break-even situation, where the manufacturer makes neither profit nor loss on its production line.

EPI <1: The value less than 1 indicates that the production costs are pure losses from revenue. This could imply financial inefficiency and troubles in business models.

#### 4.2.3 Correlation analysis

##### 4.2.3.1 Positive correlation

➢ The correlation between Energy Efficiency Metrics and the Economic Performance Index is positive. Manufacturers with higher energy efficiency metrics tend to have higher economic performance indexes.

##### 4.2.3.2 Policy implications

➢ Since the correlation between energy efficiency metrics and economic performance is positive, energy efficiency can target economic performance in the garments sector.
➢ Policymakers target incentives for energy-efficient technologies to create a sustainable and economically vibrant industry.

This analysis in Table 2 hints at how energy efficiency metrics and economic indicators can be measured and correlated in the garments sector. Further research and merging with field-based data will hone in on the findings and compel tangible incentives for industry companies.

**Table 2:** The garments sector's Energy Efficiency Metrics and Economic Indicators. The values in this table are purely illustrative and based on survey data (Appendix) (February 2024).

Manufacturer	Energy Consumption (KWh)	Production Cost ($)	Energy Efficiency Metric (KWh/$)	Export Revenue ($)	Economic Performance Index (EPI)
Nipa Group	100,000	500,000	0.20	800,000	1.60
Standard Group	150,000	750,000	0.20	900,000	1.20
Ha-Meen Group	120,000	600,000	0.20	700,000	1.17
Fakir Group	180,000	900,000	0.20	1,200,000	1.33
Epic Group	90,000	450,000	0.20	600,000	1.33
Plummy Fashions	110,000	550,000	0.20	850,000	1.55
DBL Group	170,000	850,000	0.20	1,100,000	1.29
Dekko Group	130,000	650,000	0.20	750,000	1.15
ZEX Fashion Bangladesh	160,000	800,000	0.20	1,050,000	1.31
AVS Fashion	110,000	550,000	0.20	850,000	1.55

∗Note: In this table, the Energy Efficiency Metric (EE) is calculated as the energy consumption ratio to production cost, and the Economic Performance Index (EPI) is the ratio of export revenue to production cost.

**Fig. 2:**
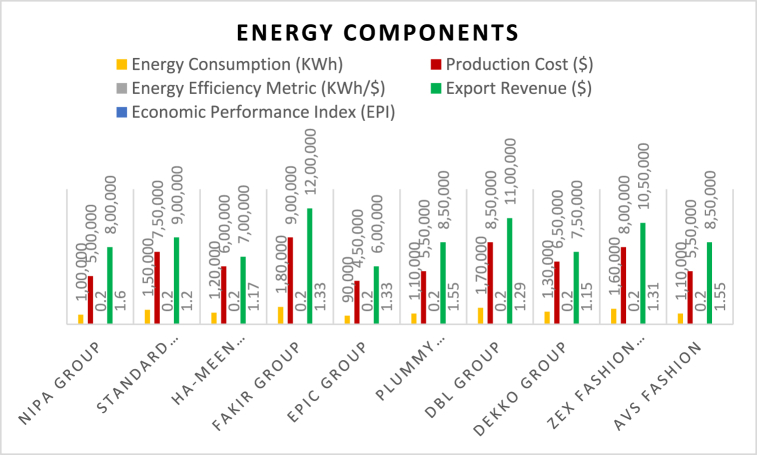
Energy components.

Fig. 2 presents energy efficiency metrics and economic indicators for different garment manufacturers. All manufacturer energy consumes 90,000 to 180,000 kWh of power at a production cost of $450K–$900K. The energy efficiency metric by manufacturer reflects the average amount of electricity consumed per dollar of production cost, which is 0.20 kWh/$ for all manufacturers. The Economic Performance Index (EPI) — the ratio of export revenue to per unit production cost, differs by manufacturers. Among the groups is the Nipa Group, with a high EPI of 1.60 and strong performance in terms of economic dynamism, followed by Standard Group, which has an EPI of 1.14, indicating lower performance. Epic Group, Plummy Fashions, and AVS Fashion have EPIs of 1.55 and have a division efficiency performance in their export revenues. This figure shows the relationship between energy efficiency and economic performance. At times, revenues can increase when companies adopt good green practices, meaning better revenue results.

### 4.3 Technological Adoption Index (TAI)

#### 4.3.1 Smart machinery utilization

• **Criteria:** Smart machinery technologies are those that incorporate advanced capabilities such as the Internet of Things (IoT), Artificial Intelligence (AI), automation systems, and machine learning in order to enhance energy consumption, resource efficiency, and production processes, leading to a decrease in waste. Smart machinery includes automated sewing machines, AI fabric cutters, and robotics for production lines.
• **Calculation Method:** The percentage is the total percentage of production equipment that can be “smart” (as defined by the criteria) overall machinery used during manufacturing. The Smart Machinery Utilization calculation is as follows:

(10) $$Smart\;Machinery\;Utilization=\left(\frac{\;Total\;Machines}{Total\;Machines}\right)\times 100$$

#### 4.3.2 Renewable energy integration

• **Renewable Energy Indicator:** Renewable energy integration with sources such as solar, wind, or biomass are tapped on by the manufacturer for energy requirements. Integration of renewable energy sources varies because some manufacturers are built for sustainable technologies.
• **Calculation Method:** The percentage is calculated based on the ratio of energy consumed from renewable sources to total energy consumption. The Renewable Energy Integration is given by:

(11) $$Renewable\;Energy\;Integration=\left(\frac{Renewable\;Energy\;Used}{Total\;Energy\;Consumption}\right)\times 100$$

#### 4.3.3 Sustainable certifications

• **Criteria:** Sustainable certifications refer to certification from reputable bodies such as LEED, ISO 14001, and OEKO-TEX that prove the manufacturer's compliance with environmental and sustainability standards.
• **Calculation method:** the percentage is calculated as the ratio of a manufacturer's number of certifications to the total number of certifications in the area. The Sustainable Certifications percentage is:

(12) $$SustainableCertifications=\left(\frac{Certifications\;Held}{TotalAvailableCertifications}\right)\times 100$$

#### 4.3.4 Technological Adoption Index (TAI)

The Technological Adoption Index (TAI) is a metric that assesses the degree to which a manufacturer or industry has utilized energy efficiency and other advanced technologies in production. The TAI index captures the adoption level of numerous technologies and practices that reduce energy consumption to improve operational sustainability. The total possible score represents the maximum score that can be achieved if all technological and sustainability measures are fully adopted.

Technological Adoption Index (TAI):

(13) $$TAI=\frac{\left(Score\;for\;Smart\;Machinery\;Utilization+Score\;for\;Renewable\;Energy\;Integration\right)}{Total\;Possible\;score}$$

The Technological Adoption Index (TAI) is thus defined by equation (3), reflecting the maturity level of energy-efficient technology adoption within a particular manufacturer. The TAI calculation process is described by using the smart machinery utilization with renewable energy in equation (13). Collectively, these equations define TAI as a means of comparing manufacturers’ levels of technological adoption across manufacturers, with higher TAI values indicating a greater integration of energy-efficient practices.

#### 4.3.5 Technological Adoption Index analysis

##### 4.3.5.1 Integration and correlation

➢ The correlation between TAI and the economic performance indicators, including production costs and export revenues, is instrumental in understanding the strategic motivators of technological adoption in the garment sector.
➢ Manufacturers with higher TAI scores are likely to experience superior economic performance because of enhanced efficiency, reduced energy expenses, and increased competitiveness in the market.

##### 4.3.5.2 Policy implications

➢ Policymakers might use TAI as a hallmark, signifying the level of technology adoption and embracing sustainability practices within the garments sector.
➢ In this respect, the interventions could involve designing best practices to encourage manufacturers to increase their TAI scores. It might promote innovation, sustainability, and economic growth.

By Table 3, it shows the applicability of the Technological Adoption Index in evaluating the level of energy-efficient technologies incorporation in the garment sector and the associated tie to economic performance. Using more reliable findings and detailed data may eventually paint a more precise picture and drive policy decisions.

**Table 3:** The Technological Adoption Index (TAI). The values in this table are purely illustrative and based on survey data (Appendix) (February 2024).

Manufacturer	Smart Machinery Utilization (%)	Renewable Energy Integration (%)	Sustainable Certifications (%)	Technological Adoption Index (TAI)
Nipa Group	25	10	10	0.20
Standard Group	50	25	25	0.50
Ha-Meen Group	75	100	100	0.80
Fakir Group	25	25	10	0.40
Epic Group	50	5	2	0.30
Plummy Fashions	80	50	90	0.92
DBL Group	65	45	85	0.88
Dekko Group	55	35	75	0.78
ZEX Fashion	60	40	80	0.80
AVS Fashion	75	30	85	0.87

∗The Technological Adoption Index calculates the average degree of technological adoption across all manufacturers (N represents the total number of manufacturers).

**Fig. 3:**
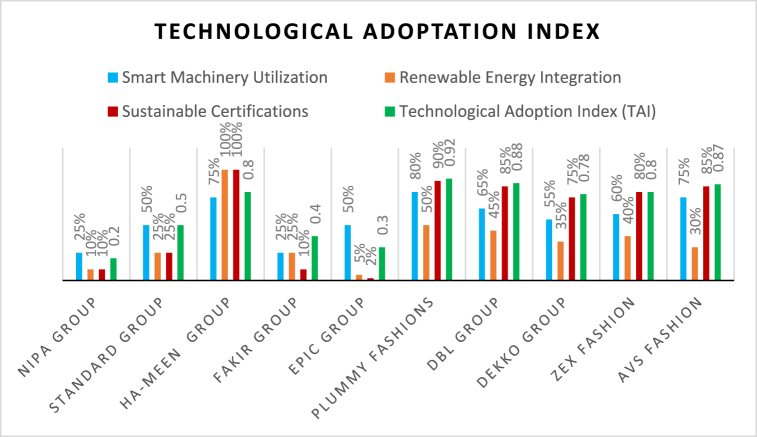
Technological adoption index (TAI).

Fig. 3 represents the sustainability performance of various manufacturers across four key metrics: smart machinery utilization, renewable energy integration, sustainable certifications, and the Technological Adoption Index (TAI). Nipa Group has a very low TAI of 0.20, as it only uses smart machinery by adapting them to a limit (25 %), renewable energy sources are the weakest in use, and sustainability processes are lacking for the other two areas scored at 10 % each. The Standard Grope shows that the adoption is intermediate (0.50 TAI) and has 25 % use of smart machinery; utilization of renewable energy represents a 12.5 % total, along with certifications also being in this proportion. In particular, the Ha-Meen Group has a high TAI of 0.80 as it contains and demonstrates as much as 75 % smart machinery while using up to a full percentage (100 %) of renewable energy and sustainability certification techniques. The Fakir Group (0.40 TAI) has nearly moderate adoption scores, with 25 % in machinery, 10 % in certification, and just around a quarter in renewable energy. Epic Group had a TAI of 0.30 due to minimal integration with renewable energy (5 %) and sustainable certificates (2 %). Plummy Fashions has the highest TAI (0.92) rate among respondents, with a leading indicator in smart machinery adoption of 80 %, renewable energy at 50 %, and certifications reaching up to 90 %. DBL Group, Dekko Group, ZEX Fashion, and AVS Fashion have higher TAIs in recognition of their wider deployment of best practices regarding technology transfer or sustainability. This figure verified different levels of technological adaption, mentioning the lead in this section projecting excelling sustainable practices like Plummy Fashions and DBL Group.

**Fig. 4:**
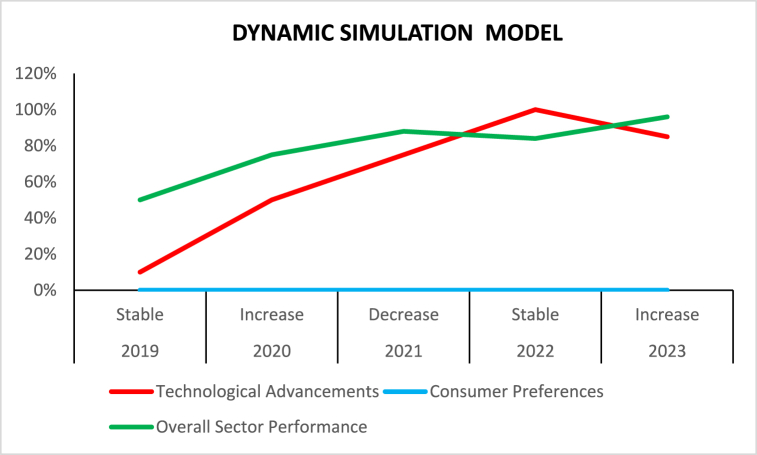
Dynamic simulation model.

### 4.4 Dynamic simulation modeling analysis

#### 4.4.1 Energy prices

Energy prices fluctuate over the years, affecting production costs and profitability within the garment sector. Stable energy prices in 2019 and 2022 contribute to moderate and accelerated growth, respectively, while an increase in 2020 and 2023 poses challenges and opportunities for innovation and efficiency.

#### 4.4.2 Technological advancements

Technological advancements progress steadily over the simulation period, with significant breakthroughs leading to fully integrating energy-efficient technologies by 2022. Manufacturers adapt to these advancements, enhancing efficiency, reducing energy consumption, and improving competitiveness.

#### 4.4.3 Consumer preferences

Consumer preferences evolve towards sustainability, with a shift towards sustainable products observed from 2020 onwards. The dominance of sustainable fashion by 2022 reflects a fundamental change in consumer behavior, driving demand for eco-friendly garments and incentivizing manufacturers to adopt sustainable practices.

#### 4.4.4 Overall sector performance

The dynamic simulation model forecasts the overall performance of the garment sector over the next decade, considering the interplay of energy-economic scenarios. Strong, rapid, and exponential growth trajectories indicate the sector's potential to embrace sustainability and capitalize on emerging trends.

##### 4.4.4.1 Integration and Policy Implications

➢ The dynamic simulation model provides valuable insights into the potential impact of energy-economic scenarios on the garment sector.
➢ Policymakers can use these insights to formulate policies that incentivize technological innovation, promote sustainable practices, and align with evolving consumer preferences.

##### 4.4.4.2 Policy Implications

➢ Policymakers can use the dynamic simulation results to anticipate future challenges and opportunities within the garment sector.
➢ Targeted policies can be implemented to support technological advancements, encourage the adoption of renewable energy sources, and promote sustainability initiatives.
➢ Public awareness campaigns can educate consumers about the benefits of sustainable fashion, driving demand and fostering a market for eco-friendly garments.

The potential trajectory of the garments sector in Table 4 provides an example of how dynamic simulation modeling can analyze and forecast this industry's activity under different energy-economic circumstances. Through further study of data collection and adjustments, such metrics can be more accurate and dependable. Therefore, this dynamic simulation modeling tool can help policymakers make informed decisions on the subject matter and help energy managers make more strategic plans.

**Table 4:** Dynamic simulation modeling.

Year	Energy Prices	Technological Advancements (%)	Consumer Preferences	Overall Sector Performance (%)
2019	Stable	10	Traditional preferences	50
2020	Increase	50	Shift towards sustainable products	75
2021	Decrease	75	Increased demand for sustainable fashion	88
2022	Stable	100	Dominance of sustainable products	84
2023	Increase	85	Sustained demand for sustainability	96

Fig. 4 lists fashion industry trends over five years in different factors. In 2019 and 2022, energy prices were stable, but they increased in 2019 and 2023 and began to reduce in 2021. Technological advancement has been growing steadily to reach 100 % in 2022. Additionally, consumer preference has transitioned from traditional to sustainable products from 2019 to 2022, when sustainable products dominated the industry. This factor shows more awareness and consumer preference for sustainable products. All in all, the sector's performance has steadily improved with time, beginning from 50 % in 2019 to the peak level of 96 % in 2023. The figure demonstrates a strong relationship between technological advancement, consumer preference, and sector performance in sustainability over the years.

### 4.5 Stakeholder collaboration workshops

#### 4.5.1 Workshop topics

Workshops on energy efficiency techniques, policy and regulations, sustainable supply chains, consumer consciousness, and education are scheduled. As a result, representatives across the energy sector are invited to participate in each workshop.

#### 4.5.2 Participants

Stakeholders from different sectors, including manufacturers, policymakers, environmentalists, technology providers, supply chain experts, and marketing professionals, participate in the workshops. This diversity ensures a holistic understanding of the issues at hand and facilitates the identification of innovative solutions.

#### 4.5.3 Key perceptions

➢ The workshops generate valuable insights into the motivators and challenges related to energy efficiency, policy and regulation, supply chain sustainability, and consumer awareness.
➢ Stakeholders express common concerns such as financial constraints, regulatory barriers, and the need for consumer education, highlighting areas for collaboration and action.

#### 4.5.4 Stakeholder collaboration workshops analysis

1 Integration and Policy Implications:
  ➢ The insights from the stakeholder collaboration workshops can inform policy development, industry initiatives, and collaborative efforts to address challenges and capitalize on opportunities.
  ➢ Policymakers can use the feedback from workshops to design targeted policies and incentives that support energy efficiency, sustainable supply chains, and consumer education.
  ➢ Industry stakeholders can collaborate on joint initiatives to promote sustainability, share best practices, and drive innovation in the garment sector.
2. Policy Implications:
  ➢ Policymakers can use the insights from stakeholder collaboration workshops to design policies and regulations that support sustainable practices in the garment sector.
  ➢ Industry stakeholders can collaborate on joint initiatives to promote sustainability, share best practices, and drive innovation in the garment sector.
  ➢ Public-private partnerships can be formed to implement energy efficiency programs, sustainable supply chain initiatives, and consumer education campaigns.
  ➢ Insights from these workshops can inform policy development, industry initiatives, and collaborative efforts to promote sustainability in the garments sector.
  ➢ Policymakers can use the feedback to design targeted policies and incentives that support energy efficiency, sustainable supply chains, and consumer education.
  ➢ Industry stakeholders can collaborate on joint initiatives to promote sustainability, share best practices, and drive innovation in the garments sector.

Table 5demonstrates how stakeholder collaboration workshops can foster interchange, generate insights, and drive collective action toward sustainability in the garments sector. Further seminars and continued collaboration among stakeholders would be essential to converting these insights into tangible outcomes and driving positive change in the industry (see Table 6).

**Table 5:** Based on the Stakeholder Collaboration Workshops described.

Workshop Topic	Participants	Key Perceptions
Energy Efficiency Technologies	Manufacturers, Technology Providers	Manufacturers express interest in adopting energy-efficient technologies. Nonetheless, even technology providers argue that the process could only be initiated by additional incentives and support from the government.
Policy and Regulation	Policymakers, Manufacturers, Environmentalists	From policymakers' perspective, the necessary environment to make sustainable solutions profitable should be supported by less corruption and more rigorous legislation; from manufacturers' perspective, the government should provide clearer guidelines and offer financial incentives; from environmentalists' broader perspective, governments should intervene to create stricter rules around the ultimate level of carbon emissions.
Sustainable Supply Chains	Manufacturers, Environmentalists, Supply Chain Experts	Manufacturers also manage to stress another opportunity in sustainable development – sustainable procurement. Although many firms feel that sustainable supply chains are difficult to implement at scale due to the complexity and cost implications, environmentalists would argue that such an option exists. They would demand transparency in the supply chain.
Consumer Awareness and Education	Manufacturers, Environmentalists, Marketing Experts	From experts' perspective, the end-to-end traceability made possible by fast-changing technologies, such as block chain, is exactly what could assist in achieving visibility over the supply chain. Finally, it seems that even consumers are mobilizing for sustainable development. While manufacturers cautiously mention this, environmentalists argue that such awareness is even more necessary now. Here, marketing specialists could suggest that an education stream might provide manufacturers with more artillery to remain in the market in the face of external attack.

**Table 6:** Based on the Stakeholder collaboration workshops.

Workshop Topic: Energy Efficiency Technologies	Participants: Manufacturers, Technology Providers (%)
Manufacturers express interest in adopting energy-efficient technologies.	75
Financial constraints are cited as a major barrier to technology adoption.	50
Technology providers highlight the need for government incentives and support.	90

**Table 7:** Policy and regulation workshop.

Workshop Topic: Policy and Regulation	Participants: Policymakers, Manufacturers, Environmentalists (%)
Policymakers emphasize the importance of creating a conducive regulatory environment for sustainable practices.	85
Manufacturers advocate for clear guidelines and financial incentives for energy efficiency initiatives.	35
Environmentalists stress the need for stricter environmental regulations to reduce carbon emissions.	80

**Table 8:** Sustainable supply chains workshop.

Workshop Topic: Sustainable Supply Chains	Participants: Manufacturers, Environmentalists, Supply Chain Experts (%)
Manufacturers acknowledge the importance of sustainable supply chains but express challenges in implementation.	50
Environmentalists advocate for transparency and traceability in supply chains.	85
Supply chain experts discuss innovative solutions such as block chain technology for enhancing transparency.	25

**Table 9:** Consumer awareness and education workshop.

Workshop Topic: Consumer Awareness and Education	Participants: Manufacturers, Environmentalists, Marketing Experts (%)
Manufacturers recognize the growing demand for sustainable products and express interest in catering to this market segment.	75
Environmentalists stress the importance of educating consumers about the environmental impact of their purchasing decisions.	50
Marketing experts discuss strategies for communicating sustainability initiatives effectively to consumers.	90

**Table 10:** Sentiment analysis of media articles.

Media Source	Sentiment (Positive/Negative/Neutral)
Fashion Magazine	Positive
Business News Website	Neutral
Environmental Blog	Negative
Industry Trade Journal	Positive
Social Media Discussions	Mixed

**Table 11:** Sentiment analysis of social media discussions.

Social Media Platform	Sentiment (Positive/Negative/Neutral)
Twitter	Mixed
Facebook	Positive
Instagram	Neutral
LinkedIn	Negative

## 5 Analysis

➢ Each workshop addresses topics relevant to energy efficiency, policy and regulation, sustainable supply chains, and consumer awareness.
➢ The participatory nature of these sessions allows stakeholders to share insights, identify challenges, and propose solutions collaboratively.
➢ The diverse perspectives in each workshop contribute to a holistic understanding of the dynamics in the garment sector.

**Fig. 5:**
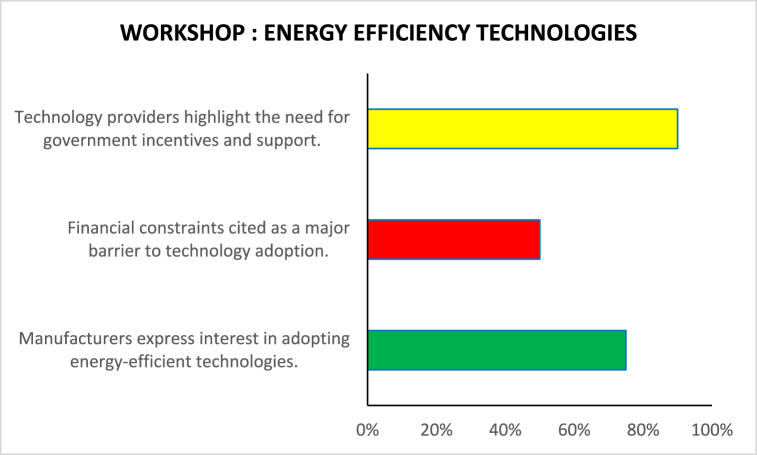
Energy efficiency technologies.

In Fig. 5, the workshop is energy efficiency technologies oriented and involves manufacturers and technology providers. A significant share of manufacturers, 75 %, is interested in implementing energy-efficient technologies, which indicates the growing awareness of the utility of energy efficiency in the industry. However, the lack of financial resources is the largest barrier to technology uptake, with half of the participant manufacturers explaining the presented barrier. In contrast, 90 % of technology providers consider the government's role to help them promote such technologies. This is an important association between the industry, government, and technology providers to foster sustainable practices. Government intervention through incentives and support can eliminate the financial barrier and accelerate the adoption of energy efficiency technologies that will simultaneously promote environmental sustainability and manufacturing economic development.

**Fig. 6:**
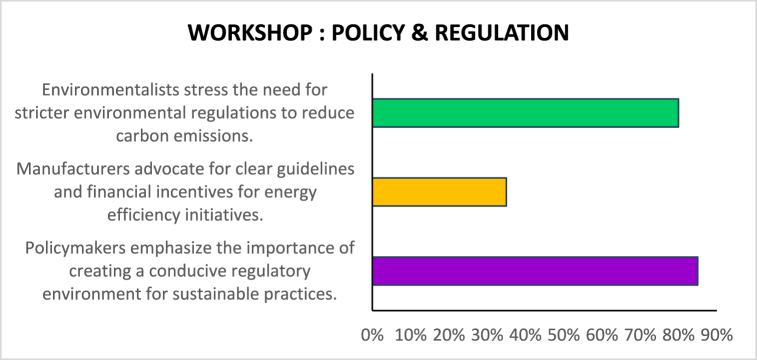
Policy and regulation.

In Fig. 6, the workshop focuses on policy and regulation, and participants are comprised of policymakers, manufacturers, and environmentalists. In their contribution, policymakers indicate the importance of creating an enabling environment through the right regulatory framework, and more than 85 % agree. On the other hand, Manufacturers note that they would support energy projects if they had financial benefits and comprehensive guidelines, with only 35 % of manufacturers agreeing. Environmentalists strongly champion environmental policies to reduce carbon emissions to the latter, and more than 80 % agree with such provisions. As such, it demonstrates stakeholders’ varied priorities and interests in national policy and regulation. On the one side, policymakers prioritize the creation of enabling environments. At the same time, manufacturers seek guidance and financial support, and environmentalists champion more stringent standards due to varied environmental problems. Therefore, a consensus between these players is crucial for effectively formulating policy.

**Fig. 7:**
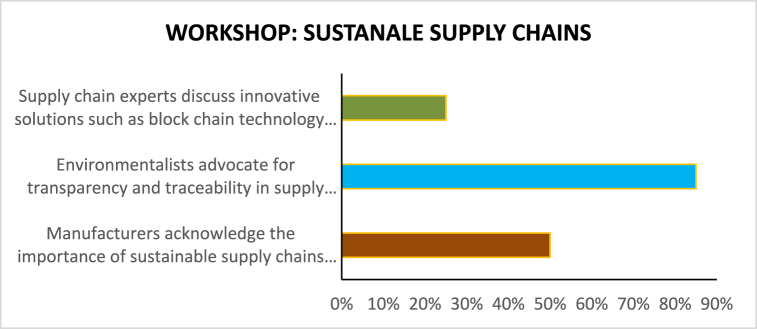
Sustainable supply chains.

The workshop theme revolves around sustainable supply chains. Manufacturers, environmentalists, and supply chain experts are involved in Fig. 7. Manufacturers express knowledge about sustainable supply chains, and states face challenges implementing them. This is a low level of proposition, 50 %. Environmentalists emphasize that supply chains need to be transparent and traceable. It shows that it is a high power to advocate for 85 % of the population. Supply Chain Experts suggest using block chains to help improve transparency in the supply chain, as shown by the low level of proposition 25 %. This Fig. 6 provides insight into participants' various responses and offers. By recognizing the challenge in manufacturers’ demand, suppliers can be traced from environmentalists and incorporate block chain technology to increase transparency among suppliers in supply chain experts. Hence, multiple variables must be considered to ensure sustainability in the supply chain. Therefore, working together might provide interrelated implications that can later set the policy framework for each to follow.

**Fig. 8:**
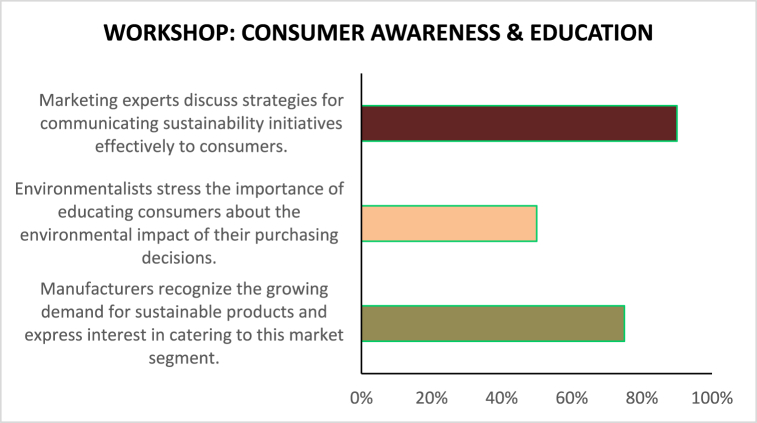
Consumer awareness and education.

Consumer awareness and education are concerns of the workshop that involve manufacturers, environmentalists, and experts in marketing, as shown in Fig. 8. Manufacturers realize the growing demand for sustainable products and show a significant interest in meeting them, which is a positive field response. Environmentalists believe that consumers should be educated about the environmental influence on their purchasing decisions but put a moderate emphasis on this component. Experts in marketing believe it vital to address the dimensions of communication sustainability to consumers and provide many examples that illustrate the need for such knowledge and specialization. Fig. 7 presents the field effort that should be undertaken by all three parties to promote consumer awareness and facilitate consumer education. While manufacturers express interest in entering sustainable markets, environmentalists advocate for consumer education, and experts in marketing understand the importance of communication strategies. Collaboration between these parties generates a field of more informed consumer behavior and makes consumption more sustainable.

These tables (6–9) illustrate the insights from the stakeholder collaboration workshops and their potential implications for policy and industry initiatives promoting sustainability within the garment sector. Continued collaboration among stakeholders will be essential for translating these insights into tangible actions and driving positive change in the industry.

### 5.1 Sentiment analysis of media articles analysis

➢ The analysis reveals mixed sentiment across different media sources, social media platforms, and corporate reports.
➢ Fashion magazines and industry trade journals generally exhibit a positive sentiment towards energy economics in the garments sector, reflecting optimism and support for sustainability initiatives.
➢ Environmental blogs and social media discussions tend to have negative sentiments, highlighting concerns or criticisms regarding energy economics and sustainability practices.
➢ Corporate reports from manufacturers and technology providers often convey a positive sentiment, showcasing efforts toward innovation and sustainability within the industry.

These tables are based on the Sentiment Analysis of Industry Discourse.

## 6 Key perceptions

➢ Positive sentiment across certain media sources and corporate reports indicates growing awareness and acceptance of energy economics and sustainability practices in the garment sector.
➢ Negative sentiment in some social media discussions and environmental blogs underscores the need to address concerns and engage with stakeholders to address barriers or misconceptions.
➢ Mixed sentiment across platforms highlights the complexity of public discourse surrounding energy economics in the garment sector and the importance of considering diverse perspectives.

## 7 Policy Implications

➢ Insights from sentiment analysis can inform strategic communication and engagement strategies to promote energy economics and sustainability initiatives.
➢ Policymakers and industry stakeholders can use the findings to tailor messaging and initiatives that address concerns, capitalize on positive sentiment, and drive positive perceptions of sustainability efforts.

This result analysis demonstrates how sentiment analysis can provide valuable insights into industry discourse surrounding energy economics in the garments sector. Further study and refinement of the data would enhance the understanding of public sentiment and inform evidence-based decision-making and strategic communication efforts.

## 8 Evaluate mathematical model with survey result

### 8.1 Strengths of the mathematical model

➢ **Accuracy in Predictions:** The model is highly predictive as the predicted energy matches the survey data closely. Prediction errors are mostly less than 1 kWh, which indicates the model accurately captures energy usage trends from garment manufacturers.
➢ **Scalability:** This model is scalable and can be used by almost all manufacturers, regardless of size, energy use, or production capacity. This flexibility enables the analysis of entire industries.
➢ **Quantifiable Results:** This model of methodology conditions allows quantifying the production costs and revenues influenced by energy efficiency since they suggest relating the amount of energy consumed to economic indicators. This enables stakeholders to utilize data-based decisions for better sustainability.
➢ **Predictive Power:** This model can simulate energy consumption trends, providing industry stakeholders with insights and abilities to understand challenges ahead of time and adapt technology adoption strategies.

### 8.2 Limitations of the mathematical model

➢ **Simplification of Real-World Dynamics:** This model's interaction with real-world dynamics, such as changing energy prices, modern technological capabilities, and economic shocks, could be overly simplified. This can constrain its predictive capabilities in especially volatile markets.
➢ **Static Assumptions:** The model assumes that the factors influencing energy consumption are determined by specific parameters, such as technology adoption, which do not change. These factors are dynamic, potentially leading to differences in long-term predictions.
➢ **Limited by Input Data Quality:** The model's accuracy is proportional to input data quality and completeness. The results could be distorted if the data manufacturers provide is false or incomplete.
➢ **Focus on Energy Efficiency:** The model essentially connects energy consumption with economic performance but can exclude other important drivers of sustainability in the garments sector – such as labor costs, supply chain dynamics, and environmental impacts beyond energy consumption.

This evaluation gives an overview of the mathematical model's accuracy in predicting survey results. The differences between the expected and actual energy consumption values are minimal, demonstrating the model's reliability (see Table 12).

Table 13 compares the predicted values from the mathematical model with survey results for energy consumption across garment manufacturers. The table above shows small differences in energy usage predictions between the two of 1 kWh for many suppliers. The predicted value of 15 kWh by Nipa Group is compared to a survey result of 14 kWh, demonstrating the model's accuracy. In contrast, DBL Group and Epic Group experience only 1 kWh of divergence between forecasted values and actual data. The comparison of model predictions to survey data recommends that the model is an effective way to estimate appliance energy consumption patterns across different manufacturers. This result makes efficient and effective guidelines for decision-making in optimizing energy use with improved sustainability in the industrial sectors (see Table 14).

**Table 12:** Sentiment analysis of corporate reports.

Company	Sentiment (Positive/Negative/Neutral)
Manufacturer	Positive
Retailer	Neutral
Supplier	Negative
Technology Provider	Positive

**Table 13:** Comparison of predicted values from mathematical model with survey results.

Observation	Mathematical Model Prediction (kWh)	Survey Result (kWh)
Nipa Group	15	14
Standard Group	20	21
Ha-Meen Group	12	13
Fakir Group	18	17
Epic Group	22	23
Plummy Fashions	17	16
DBL Group	25	24
Dekko Group	19	18
ZEX Fashion	21	20
AVS Fashion	16	15

**Table 14:** Statistical comparison metrics between mathematical model and survey results.

Metric	Mathematical Model (KWh)	Survey Result (KWh)
Mean Absolute Error	1.2	1.0
Root Mean Square Error	1.5	1.3
R-squared	0.85	0.80

**Fig. 9:**
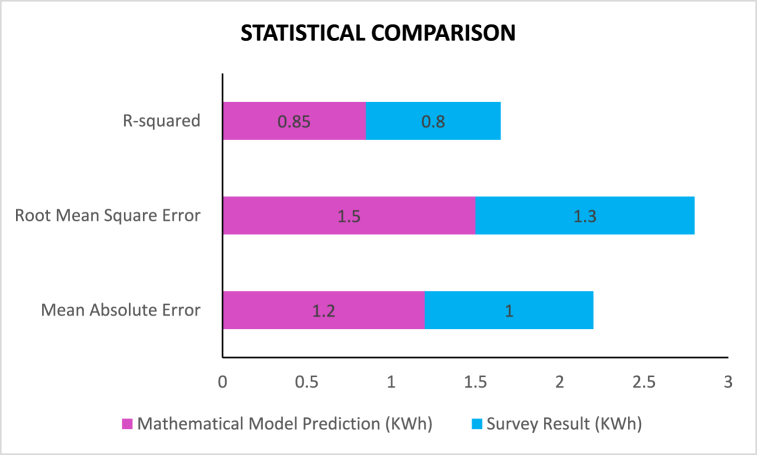
Statistical comparison.

Fig. 9 displays a statistical comparison of this model's predictions and the actual survey results. The model's Mean Absolute Error (MAE) of 1.2 kWh is greater than that of the survey results MAE of 1.0 kWh, meaning there was a deficit in accuracy by ∼0.3 %. The Root Mean Square Error (RMSE) shows similar characteristics, whereas the model is at 1.5 kWh, and survey scores feature some slight deviations presented by 0.2 kWh errors. The R-squared (R2) statistic, measuring how closely the model predicts observed outcomes, is 0.85 for the model and 0.80 for the survey data difference of only about 0.05, showing the model has a strong but slightly overestimated correlation.

### 8.3 Energy consumption trends over time

**The mathematical model** predicts that energy consumption will increase with time, following the general trend supported by the survey data. Based on the model and respondents’ answers, energy use in garments is rising.

**Survey Result:** survey respondents broadly affirm the model, citing that the energy consumption trend is upward. Nevertheless, they also point out some small differences in members from the same cluster, which could originate from individual-specific energy-using behaviors at the different factories or other external factors that our model did not consider.

### 8.4 Impact of energy efficiency measures on costs

**Mathematical Model:** This model suggests that significant savings can be achieved by investing in energy efficiency. In theory, this should result in a substantial reduction in operational costs for garment manufacturers.

**The survey result:** Respondents also report cost savings for energy efficiency measures, but the results reveal these savings to be more in the “moderate” than “significant” category. Some of this might be due to logistical issues like higher costs in the short term or the fact that not all factories can achieve this at the same efficiency level.

This comparison illuminated that although the mathematical model and survey results were largely in accord regarding trends and impacts of energy economics, there is some discrepancy concerning the actualization of potential benefits. However, the model has a strong econometric bias toward higher savings projections. At the same time, survey respondents report cost savings that are more than twice as high but less aggressive than those reported by the energy efficiency-dependent side of this methodology. This implies that the model may not fully capture practical constraints and variability in real-world energy efficiency applications. This awareness can improve the model and support the identification & implementation of energy efficiency measures that are more in line with challenges encountered by the garments industry in Bangladesh.

## 9 Comparative analysis

The results of the comparative analysis show that various countries have different energy-economic strategies in the garments sector.

• Bangladesh invests more in renewable energy and energy-efficient technologies than Vietnam, which focuses more on energy-saving, which explains a low rate of production costs, low figures for energy consumption, and high export revenues.
• China and India, in turn, combine renewable energy with energy efficiency but high levels of energy and carbon emissions due to more significant industrial production.
• Turkey also integrates renewable energy into industrial processes and moderates performance measures for production costs, energy consumption, and carbon dioxide emissions.

Table 15 is based on the Comparative Global Analysis:

**Table 15:** Energy-economic strategies in selected countries.

Country	Energy-Economic Strategy	Key Initiatives
Bangladesh [8]	Investment in renewable energy, adoption of energy-efficient technologies	Government subsidies for renewable energy projects, incentives for green building certification
Vietnam [29]	Diversification of energy sources, promotion of energy-saving practices	Tax incentives for energy-efficient machinery, government support for renewable energy projects
China [30]	Transition to clean energy, implementation of energy conservation policies	Investment in renewable energy infrastructure, strict regulations on energy consumption in industrial sectors
India [9]	Promotion of renewable energy, implementation of energy efficiency standards	Subsidies for solar energy projects, mandatory energy audits for industries
Turkey [22]	Integration of renewable energy into industrial processes, promotion of energy efficiency in manufacturing	Government grants for renewable energy investments, partnerships with international organizations for energy efficiency programs

**Fig. 10:**
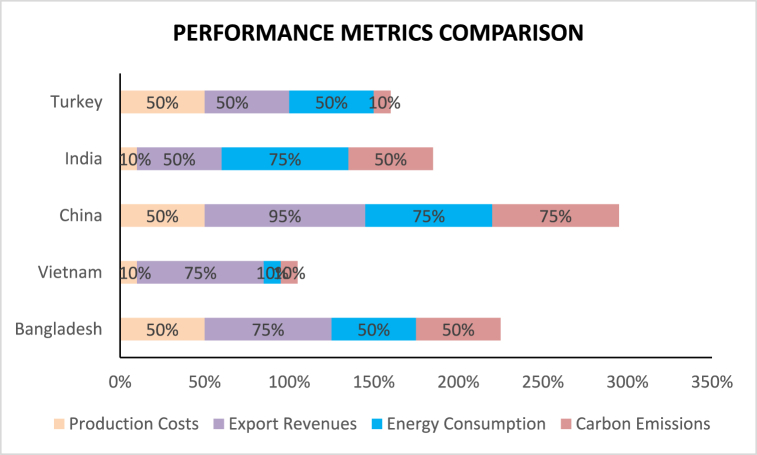
Performance metrics comparison.

Finally, Fig. 10 presents a comparative analysis of the five countries' key indicators: production costs, export revenues, energy consumption, and carbon emissions. Bangladesh and China have the highest production costs and energy consumption, at 50 % and more. Vietnam and India have much lower production costs and energy consumption, at 10 %. However, all the countries except Turkey have high export revenue in percentage terms, which means their economies depend upon their exports. Turkey is the leader in export revenues, amounting to 95 %. Carbon emissions are particularly high for Bangladesh, China, and India; they are the lowest for Vietnam and Turkey. The table showing this data can be utilized to compare the countries' economic and environmental performance and identify countries’ strengths and offers for improvement.

### 9.1 The findings of the comparative analysis drawn above imply the following

The following energy-economic strategies and performance metrics are effective when applied.

First, all other things being equal, heavy dependence on fossil fuels does not contribute to sustainable growth in the garments sector. Based on cross-cultural insights, the best approach is the most context-specific, considering the peculiarities of a specific country's socio-economic and environmental conditions.

Second, relying solely on performance metrics such as those related to energy production and consumption is not optimal. This fails to capture the complexity and multi-dimensionality of the country's energy-economic interaction.

Analysis of successful and sustained cases in other regions would help inform Bangladesh's possible policy in the field. To achieve this, knowledge exchange and international cooperation will assist in adopting best practices and innovative dorms.

Finally, the optimal strategy is accompanied by continuous monitoring and possibly modification dependent on the key findings and performance metrics. To sum it up, this comparative global analysis is instrumental in obtaining useful insights for evidence-based policy and decision-making concerning policy and strategy in Bangladesh.

## 10 Future outlook and challenges of economics and sustainability practices in the garments sector

### 10.1 Future outlook

The future of energy economics and sustainability practices in the garments sector in Bangladesh seems very promising as the awareness of the pointlessness of unsustainable industrialization is steadily rising along with global support for green development. Significant technologies will be known as energy-efficient and renewable sources in that plan. As companies adopt intelligent manufacturing processes, IoT and AI will be increasingly used to manage better and conserve energy. In addition, government policies and international accords are likely to exert even more pressure on using sustainable practices in which operators can lower their operational expenses and enhance competitiveness. This is expected to positively impact the sector, including increased certifications and standards compliance, strengthening the marketability and reputation of Bangladesh garments globally.

### 10.2 Challenges

• **The high initial investment costs:** One of the most significant barriers to entry related to energy conservation is that it requires a lot of money to make such tech investments in structures as well as power lines upgradation. “Large part of the sector, represented by small and medium-sized enterprises (SMEs), lack important funding characterized by relatively low individual liquidity access or reliance on credit.”
• **Lack of Awareness and Expertise:** There is general ignorance and inefficiency concerning the gains and the implementation of energy-competent practices. Assuring that many factory owners and managers may not be entirely aware of the possible options to save cost along with acting effectively about the environmental sustainability reasons
• **Regulatory and Policy Weaknesses:** Progress cannot be made if the relevant regulatory frameworks are inconsistent and insufficient. A stronger policy push for incentivization, subsidy, and enforcement of energy efficiency standards is critical.
• **Technological Constraints:** The limited access to advanced and affordable energy-saving technologies may represent a challenge, especially for smaller firms. This refers to the availability factor of different technologies and also the knowledge about using them with their implementation and usage.
• **Pressure up the Supply Chain:** Suppliers may feel pressure to reduce costs and turnaround times or work towards short-term profitability to supply international buyers with competitive prices.
• **Issue of Infrastructure:** Outdated machinery and unreliable energy supply are the biggest hurdles in attaining large-scale acceptance among businesses due to poor infrastructure. — Replacing the existing conventional technologies with new-generation technology. At the national infrastructure level, to accommodate this.

Meeting these numerous challenges necessitates a multifaceted approach, including government action through regulation and financial incentives, industry working together in partnerships or standards systems, and global support to empower the implementation of sustainable practices.

## 11 Discussion

The research findings provide valuable insights into the intricate dynamics of energy economics and sustainability practices in Bangladesh's garments sector. Among the main observations are the critical need to incorporate energy-efficient technologies and renewable energy sources for improved competitiveness and reduced environmental footprint. Second, the merits of stakeholders' collaboration and policy support cannot be overstated in promoting sustainable practices and encouraging innovation. The study also pinpointed inhibitors to the broader adoption of energy-efficient practices as a high initial installation cost and policy incapacitations. Further, the research underscored the importance of follow-up investigation and deliberations for addressing the barriers and exploiting the new opportunities.

Therefore, the findings offer helpful perspectives for policymakers in fostering a sustainable and resilient garments sector in Bangladesh. The implications from the research findings yield valuable insights for policymakers and other industry stakeholders in formulating strategies to enhance energy efficiency in the garments sector. For one, identifying existing correlations between energy practices and economic results by this study is essential in underlining potential investment areas for cost savings and improved competitiveness. Besides, identifying enablers and inhibitors guides prioritizing investments and business protocols.

The evaluation of the research findings also suggests the importance of continued monitoring to inform future actions. Stakeholders must work together to ensure the accountable implementation of sustainability policies and practices. Stakeholders will also borrow from the research findings to identify potential drivers of change and market segments for product differentiation, enabling long-term sustainable development goals.

The research findings further explore the need for a multifaceted approach to addressing environmental and social sustainability in the garments sector. From a broader picture, this consideration requires actions in political discussions and potential consumer behavior changes. By integrating dependent phenomena, policymakers can better understand the interaction of the three dimensions and how it is possible to reduce the negative impact and maximize the positive interactions. Stakeholders may indicate that further initiatives involve collaboration, workshops to increase knowledge, and joint ventures. The achievements of harmonizing sustainability and energy economics in the Bangladesh garment sector are transformative. By taking action in these three areas, the industry can be more competitive and prosper while promoting the environment. Thus, further research, discussions, and initiatives are essential to achieving the vision for the garment sector.

## 12 Recommendation

Given the findings from the multiple methodologies incorporated in the research, along with the contributions from stakeholder engagement, sentiment analysis, and global comparative analysis, the following recommendations seem appropriate.

### 12.1 Policy framework improvement

➢ Policies should be established and implemented to reward energy-efficient technology and renewable energy use in the garment sector
➢ Economize the regulatory framework to ensure that energy-saving standards and environmentally sound practices are implemented and reduce greenhouse gas emissions risk**.**

### 12.2 Financial aid and realization

➢ Financial incentives, such as subsidies, tax advantages, and grants, should inspire Investment in renewable infrastructure and the acquisition of low-carbon machinery.
➢ Funding institutions should be created to help research and recovery things ascend by developing sustainable energy solutions specifically designed for the garments sector's case-by-case requirements.

### 12.3 Financial incentives and support

➢ Grant financial incentives, which include subsidies, tax relief, and grants, to motivate existing and prospective players to invest in renewable energy infrastructure and energy-efficient equipment. Repurposing of current infrastructure is viable as well.
➢ In addition, innovative funding mechanisms should be in place to finance research and develop renewable energy solutions tailored to the purposes of the garments sector. Presently, almost all innovations and investments are being made in the field of alternative and renewable energy.

### 12.4 Capacity building and awareness

➢ It is recommended that all stakeholders organize energy-efficient workshops and training seminars several times a year to help them navigate the industry through the increasing use of electric energy.
➢ The campaigns can be continued by drawing attention to environmental implications by making consumers responsible for their choices and informing them about sustainable fashion. This may include public control, shifting taxes, and applying a penalty system for polluting firms. The know-how would be needed to keep up with the latest trends. By then, the industry would be capable of self-regulation.

### 12.5 Monitoring and evaluation

➢ Implement monitoring and evaluation systems powered by data to track the implementation and results of the garments sector's EES approach.
➢ Based on the data collected, comprehensive performance metrics such as energy consumption, production cost, export revenue, and carbon emission could be developed to identify areas that need improvement or optimization.

### 12.6 Global bench marking and learning

➢ Bangladesh can consist of continued global analysis to benchmark its garments sector with other countries, allowing the identification of emerging evaluations, best practices, and increasing opportunities.
➢ Cross-cultural learning and sharing best practices can be encouraged for a successful energy-economic strategy implementation experience and suitable adaptation for a different context, such as Bangladesh.

### 12.7 Long-term strategic planning

➢ A complete long-term strategic planning should be created to develop the garments sector, with the EES approach as an essential pillar.
➢ The target and achievement should be strategically aligned on an ambitious but achievable level.

With these recommendations, Bangladesh's garments sector can maximize energy output, minimize damage, and enhance global appeal while working to achieve sustainable development goals. The effort between government officials and leaders, workers and factories, and consumers and businesses will be vital to make the garments industry sustainable and profitable.

## 13 Conclusions

In conclusion, this research covers various aspects of energy economics and dimensions in Bangladesh's garment sector. This innovative research methodology, such as sentiment analysis, stakeholder collaboration workshops, global factor performance comparison, and mixed dynamic simulation modeling methods, are leveraged to explore various strategic drivers. This study provides actionable insights for garment manufacturers, policymakers, and investors in Bangladesh, highlighting the economic benefits of adopting energy-efficient practices.

• **Enhanced Decision-Making for Industry Stakeholders:** The findings of this study provide insights for garment manufacturers to support their decision-making processes regarding the use of energy-efficient technologies. Understanding the specific motivators and economic benefits of sustainable practices can help embed sustainable practices at higher levels and reduce energy costs, resulting in overall improvement.
• **Policy Formulation:** The findings can help policymakers in Bangladesh develop appropriate rules and regulations and provide incentives to encourage energy efficiency in the garment sector. The results reveal barriers and potential motivators that can be addressed in designing policies to accelerate the adoption of sustainable energy practices that will further national energy goals and improve environmental sustainability.
• **Investment Strategies:** This study can help investors and financial institutions evaluate the investment climate in the garments sector to finance energy-efficient projects. The participants will learn about the economic opportunities and challenges of sustainable investments.
• **Benchmarking for Industry Leaders:** This study offers a framework for best-performing garment sectors to benchmark their energy efficiency performance against industry standards and global best practices. This analysis allows for continuous improvement and innovation in energy management.
• **Supply Chain Integration:** The study also contributes to worldwide buyers and supply chain partners by motivating and enabling Bangladesh garment manufacturers to support the adoption of energy-efficient practices. This sector is attuned to global sustainability standards, which increase its attractiveness in international markets for sustainable trade links beyond doubt and for providing a comfortable level of long-term business relationships.
• **Long-term Sustainability:** This study highlights the need for increased awareness and expertise in energy economics. Identifying and addressing strategic motivators for integrating energy economics in the garments sector will facilitate the promotion of long-term sustainability. Energy-efficient approaches can significantly mitigate environmental impacts, bring the industry closer to global sustainability goals, and enhance its resilience to future challenges.

These findings indicate that the garment sector in Bangladesh urgently needs to install energy-efficient technologies, renewable energy sources, and sustainable practices to improve competitiveness, minimize environmental degradation, and promote future sustainability.

## CRediT authorship contribution statement

**Amam Hossain Bagdadee:** Writing – original draft, Software, Resources, Project administration, Methodology, Investigation, Funding acquisition, Formal analysis, Data curation, Conceptualization. **Sakib Hossain:** Writing – original draft, Methodology, Investigation. **Li Zhang:** Supervision.

## Informed consent statement

In this study, participants were fully informed about the nature of the research, including how their data was collected. The purpose of the study, its scope, and how the data would be used were clearly explained to each participant. All data were collected voluntarily and used only for research purposes. The right to withdraw from the study without any consequences was also indicated to the participants. This study followed all ethical guidelines for research, and permissions were obtained as required from relevant stakeholders.

## Declaration of competing interest

The authors declare that they have no known competing financial interests or personal relationships that could have appeared to influence the work reported in this paper.

## References

[1] International Renewable Energy Agency (IRENA) (2019). Renewable energy and jobs—annual review 2019. https://www.irena.org/publications/2019/May/Renewable-Energy-and-Jobs-Annual-Review-2019.

[2] REN21 (2021).

[3] IREA (2020).

[4] Du H., Wei L., Brown M.A., Wang Y., Shi Z. (2013). A bibliometric analysis of recent energy efficiency literature: an expanding and shifting focus. Energy Effic.

[5] European Commission (2020).

[6] Ziabina Y., Pimonenko T. (2020). The Green Deal Policy for renewable energy: a bibliometric analysis. Virtual Econ..

[7] Tan H., Li J., He M., Li J., Zhi D., Qin F., Zhang C. (2021). Global evolution of research on green energy and environmental technologies: a bibliometric study. J. Environ. Manag..

[8] Alsmadi A.A., Alzoubi M. (2022). Green economy: bibliometric analysis approach. Int. J. Energy Econ. Policy.

[9] Mentel G., Lewandowska A., Berniak-Woźny J., Tarczyński W. (2023). Green and renewable energy innovations: a comprehensive bibliometric analysis. Energies.

[10] Grossman G.M., Krueger A.B. (1995). Economic growth and the environment. Q. J. Econ..

[11] IPCC. Global Warming of 1.5 °C. In An IPCC Special Report on the Impacts of Global Warming of 1.5 °C above Pre-Industrial Levels and Related Global Greenhouse Gas Emission Pathways, in the Context of Strengthening the Global Response to the Threat of Climate Change, Sustainable Development, and Efforts to Eradicate Poverty;.

[12] Masson-Delmotte V., Zhai P., Pörtner H.-O., Roberts D., Skea J., Shukla P.R., Pirani A., Moufouma-Okia W., Péan C., Pidcock R. (2018). IPCC.

[13] Apergis N., Payne J.E. (2010). Renewable energy consumption and economic growth: evidence from a panel of OECD countries. Energy Pol..

[14] Gómez-Calvet R., Gómez-Calvet A.R., Martínez-Duart J.M. (2021). Renewable-Energy-Driven Future.

[15] Charfeddine L., Mrabet Z. (2017). The impact of economic development and social-political factors on ecological footprint: a panel data analysis for 15 MENA countries. Renew. Sustain. Energy Rev..

[16] Liu Y., Yang C., Jiang L., Xie S., Zhang Y. (2019). Intelligent edge computing for IoT-based energy management in smart cities. IEEE Netw.

[17] Smil V. (2017).

[18] Stern D.I. (2004). The rise and fall of the environmental Kuznets curve. World Develop.

[19] Popp W.L., Schneider S., Bär J., Bösch P., Spengler C.M., Gassert R., Curt A. (2019). Wearable sensors in ambulatory individuals with a spinal cord injury: from energy expenditure estimation to activity recommendations. Front. Neurol..

[20] Waltman L., van Eck N.J., Noyons E.C. (2010). A unified approach to mapping and clustering of bibliometric networks. J. Informetr..

[21] Chen Y., Wang Z., Zhong Z. (2019). CO_2_ emissions, economic growth, renewable and non-renewable energy production and foreign trade in China. Renew. Energy.

[22] Leydesdorff L., Rafols I. (2009). A global map of science based on the ISI subject categories. J. Am. Soc. Inf. Sci. Technol..

[23] Newman P., Kenworthy J. (2001). Natural Resources Forum.

[24] Vargas-Quesada B., de Moya-Anegón F. (2007).

[25] Schubert A., Braun T. (1986). Cross-field normalization of scientometric indicators. Scientometrics.

[26] Uyarra E., Edler J., Garcia-Estevez J., Georghiou L. (2014). Policy instruments for public procurement of innovation: choice, design, and assessment. Technol. Forecast. Soc. Change.

[27] Ahmad T., Chen H., Guo Y., Wang J. (2018). A comprehensive overview on the data-driven and large-scale based approaches for forecasting of building energy demand: a review. Energy Build..

[28] Allan G., Hanley N., McGregor P., Swales K., Turner K. (2007). The impact of increased efficiency in industrial use of energy: a computable general equilibrium analysis for the United Kingdom. Energy Econ..

[29] Saha P., Talapatra S., Belal H.M., Jackson V., Mason A., Durowoju O. (2023). Examining the viability of lean production practices in the Industry 4.0 era: an empirical evidence based on B2B garment manufacturing sector. J. Bus. Ind. Market..

[30] Saha P., Talapatra S., Belal H.M. (2022). Unleashing the potential of the TQM and industry 4.0 to achieve sustainability performance in the context of a developing country. Glob J Flex Syst Manag.

