# Supplementary material for: Strategic motivators for integrating energy economics into the developing garments sector: A comprehensive analysis from Bangladesh
Source: Heliyon. 2024 Nov 23;10(23):e40631. doi: 10.1016/j.heliyon.2024.e40631 (PMC11652842; doi:10.1016/j.heliyon.2024.e40631)
Supplement: Multimedia component 1 [file mmc1.docx]

**Appendix**

**Survey Questionnaire: Hybrid Quantitative-Qualitative Approach**

*Introduction:* Thank you for participating in this survey. Your responses will help us gain valuable insights into energy economics and sustainability practices in the garments sector. Please answer the following questions to the best of your knowledge and experience.

**Section 1: Quantitative Survey**

1. How could we please assess the current level of our energy efficiency in the plant-producing garments?
2. Good
3. Excellent
4. Fair
5. Poor
6. Not Applicable
7. What share of your total production expenses goes to energy?
8. Less than 5%
9. 5% - 10%
10. 10% - 15%
11. More than 15%
12. Not Sure
13. Do you utilize renewable sources of energy, like solar or heat?
14. Yes
15. No
16. Planning to in the future
17. Not Applicable
18. How do you think it impacts your production output?
19. Positive impact
20. No significant impact
21. Negative impact
22. Not Applicable
23. What are the main barriers/challenges to implementing energy-efficient practices in your facility? Select all that apply:
24. High initial investment costs
25. Lack of technical expertise/knowledge
26. Insufficient government incentives/support
27. Regulatory barriers
28. Lack of awareness among stakeholders
29. Other (please specify) __________

**Section 2: Qualitative Insights**

1. Can you indicate some specific initiatives or strategies implemented by your facility in this regard? [Open-ended]
2. What is the role of government policies/ regulations in promoting energy efficiency and sustainability in the garments sector? [Open-ended]
3. In your opinion, what are the key energy economics integrators of garment manufacturing operations?
4. Open-ended response: What challenges can arise from integrating sustainable energy practices into garment sector operations?

**Section 3: Demographic Information**

1. Open-ended response: What is the annual revenue generated by your garment manufacturing facility?
2. Small (less than $1 million)
3. Medium ($1 million - $10 million)
4. Large (more than $10 million)
5. How long has your facility been operational in the garment manufacturing industry?
6. Less than 5 years
7. 5-10 years
8. More than 10 years
9. Which region of Bangladesh is your facility located?
10. Dhaka
11. Chittagong
12. Other (please specify) __________

**Section 4: Technological Adoption and Innovation**

1. To what extent does your facility utilize smart machinery or automation, among others, to optimize energy efficiency in the production process?
2. Extensively
3. Moderately
4. Minimally
5. Not at all
6. Have you considered implementing sustainable certifications such as LEED ISO 14001, among others, in your facility to promote energy efficiency and environmental sustainability?
7. Yes, we have already implemented it.
8. Yes, we are planning to implement
9. No, not considering
10. Not sure

**Section 5: Environmental Impact and Sustainability Practices**

1. How do you measure and monitor your facility’s carbon footprint and environmental impact?
2. Regularly conduct environmental audits
3. Track energy consumption and emissions data
4. Use third-party certification programs
5. Not actively monitored
6. What sustainability initiatives, if any, does your facility undertake to reduce environmental impact aside from energy efficiency measures?
7. Waste reduction and recycling programs
8. Water conservation measures
9. Sustainable sourcing of materials
10. Employee training and awareness programs
11. Community engagement and CSR initiatives
12. Other (please specify) __________

**Section 6: Stakeholder Engagement and Collaboration**

1. To what extent do you engage with external stakeholders such as government agencies, NGOs, industry associations, and others to promote energy efficiency and sustainability in the garments sector?
2. Actively engage in collaborative initiatives.
3. Limited engagement
4. No engagement
5. Open-ended: What are the biggest benefits or challenges you meet when you work with or collaborate with an external stakeholder related to energy economics and sustainability?

**Section 7: Future Outlook and Challenges**

1. Open-ended response: How do you expect energy economics and sustainability practices in Bangladesh’s garments sector in the future?
2. Open-ended response: What are the biggest challenges restricting or creating barriers to energy-efficient practices in the garments sector?

Thank you for taking the time to respond to the survey. Sharing your answers will help gather details about energy economics and sustainability practices in the garment sector. It will help to promote change and innovative practices in this industry. Thank you for taking the survey.
